# A smartphone app for preschool wheezing and reliability of medical history collection

**DOI:** 10.1186/s13052-024-01792-w

**Published:** 2024-10-25

**Authors:** Nicola Ullmann, Adriana Fracchiolla, Alessandra Boni, Valentina Negro, Federica Porcaro, Antonio Di Marco, Salvatore Tripodi, Renato Cutrera

**Affiliations:** 1 Pediatric Pulmonology & Cystic Fibrosis Unit, Bambino Gesù Children’s Hospital, IRCCS, Piazza S. Onofrio 4, 00165 Roma, Italy; 2 Allergology Service Policliclinico Casilino, Rome, Italy

**Keywords:** Wheezing, Smartphone, Application, Medical history, Reliable, Children, Preschool, Mobile, Preschool

## Abstract

**Background:**

The use of mobile applications helps improving self-management in adolescents with asthma. However, no evidence is available for children with preschool wheezing. In addition, we have no data on the reliability of medical history collected at visits. The first aim was to assess the feasibility of a smartphone app in the management of preschool wheezing; secondly we aimed to evaluate the reliability of anamnestic data collected during face-to-face medical interviews.

**Methods:**

Children with recurrent wheezing, age between 25 and 72 months, were randomly assigned to the intervention group, provided with a smartphone app for symptoms monitoring and asthma attack treatment, or to the control group, with a written action plan. At follow-up medical history was collected and the asthma control test and a clinical questionnaire were completed. App acceptability was also explored. Respiratory symptoms, medication and utilization of healthcare resources were collected. Plus, medical information obtained from the paper questionnaires was compared with data daily recorded by the app.

**Results:**

We enrolled 85 preschool children with recurrent wheezing: 43 assigned to the intervention and 42 to the control group. The average (SD) adherence to e-Diary compilation was 60 (15)%. The acceptance and usability of the intervention was favorable as 70% and 93% of participants in the intervention arm described the app as ‘’simple and intuitive’’ at Visit1 (after 3 months from enrollement) and Visit2 (3 months later than Visit1), respectively and 95% and 98% found it useful in symptoms management. There were no significant differences between the two groups in clinical outcomes. At Visit1, the cACT median score (IQR) was 23,5 (21–25) for the control group (42 patients) and 23 (21–24) for the intervention group (43 patients). At Visit2 (41 controls and 42 in the intervention group) it was 25 (24–25) and 24 (24–25), respectively. Secondary analysis of data from the intervention group showed higher incidence of daily symptoms recorded by the app in comparison with the paper questionnaire, suggesting that collection of retrospective medical history may not be completely reliable.

**Conclusions:**

The smartphone app is usable and acceptable by families of preschool wheezers. Future controlled trial are needed to prove an impact on clinical outcomes or its efficacy in a telemedicine program. Finally a daily questionnaire could provide physicians with a more reliable clinical picture as reflected better daily asthma symptoms than the written retrospective questionnaire filled at clinical visit.

## Background

Wheezing during early life represents a common disorder, although most of the patients (60%) are expected to improve and to be symptom-free at the age of 6 years [1]. However, some children develop asthma at school age and frequency of episodes of wheezing has been identified as one of the major influencing factors [2]. Children suffering from preschool recurrent wheeze (PSW) experience twice the rate of outpatient and emergency visits and 5 times the rate of hospitalization compare to children with no wheezing [3] with direct consequences on health-care and economic resources. This underlines the importance of implementing effective strategies aimed at reducing the morbidity associated with PSW [4]. The need for continued controller treatment should be regularly assessed to determine whether adjustments to therapy are required especially in children with PSW and allergic sensitization in which the odds of response of inhaled corticosteroids (ICS) are higher [5].

To improve a self-management education, action plans have been shown to be of value in older children, but they have not been extensively studied in children ≤ 6 years [6]. In recent years many mobile asthma apps have been developed and several studies showed that smartphone apps for asthma have the potential to support self-management, quality of life and health behavior change in young people with asthma [7–9]. However, the current evidence base is not sufficient to advise clinical practitioners with regards to the use of smartphone apps for the delivery of asthma self-management programs [10]. Previous studies confirmed the potential parental insecurity in the clinical evaluation of their child and underlined the need for studies to assess the benefit of digital support in a home care setting of preschool wheezing [11].

In addition, in preschool children when allergy screening and functional tests are not always available or reliable, physicians mostly rely on medical history collection to decide the best clinical management. However, the term “wheeze” itself is already problematic with possible misunderstanding with parents reporting their children’s symptoms [12]. Levy et al. described that in only 30% of preschool children with wheeze the parent and the physician agree on the wheeze severity score showing that parents were not able reliably to judge the severity of wheeze measured objectively [13]. Moreover, in a cohort of school age children and adolescents has been shown that there is a memorization bias in parents regarding their child’s symptoms [14]. Therefore, we thought that a better understanding on the reliability of medical history information collected at time of medical visits from parents of preschool wheezers is yet another aspect that deserves further attention in order to improve the correct management of patients.

In the same cohort of a previous study [15] we conducted a randomized controlled trial with the aims: (A) to determine the feasibility and efficacy of monitoring preschool wheezing children with a mobile app; (B) to compare medical history data collected during outpatients visits every 3 months with those daily collected through an app in order to assess the reliability of retrospective collection of anamnestic information.

## Materials and methods

### Study design and population

This is a randomized controlled trial conducted at the Pediatric Pulmonology Unit of the “Ospedale Pediatrico Bambino Gesù” in Rome, from November 2019 until June 2020. During this period, Italy underwent a period of lockdown due to the Covid-19 pandemic (from March 9 until May 19).

Study participants were preschool children affected by persistent wheeze and treated with preventive low doses of inhaled corticosteroids. The inclusion criteria were children, age between 25 and 72 months, with PSW with either (a) 3 reported episodes in the previous 12 months (b) 1 oral corticosteroids cycle in the previous 6 months; (c) 1 hospitalization for wheezing exacerbations in the previous 12 months. The exclusion criteria were: (1) known anatomic malformations causing a chronic bronchial obstruction; (2) any severe chronic diseases (i.e. cancer, primary immunodeficiency); (3) intention to move away from Rome during the monitoring period. Eligible participants were approached during outpatients visits by a research coordinator and a research nurse, who obtained written consent.

**Fig. 1:**
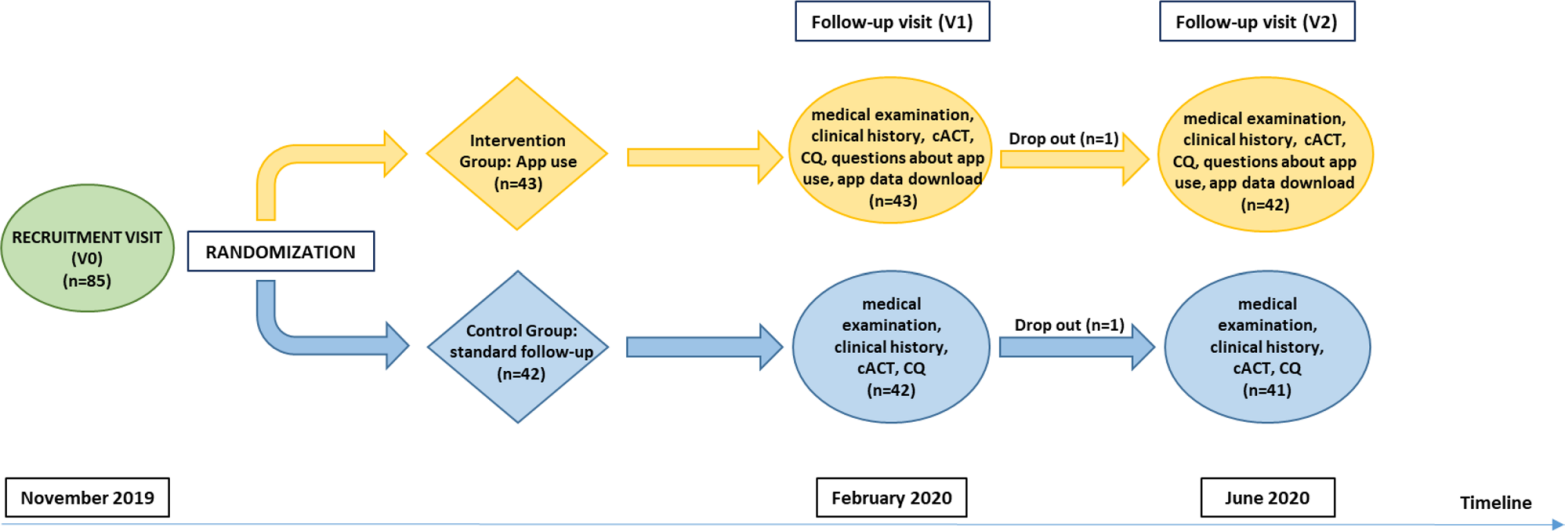
The study design. Legend: Details of the study design: recruitment, randomization and follow-up. Abbreviations: App (Smartphone Application); cACT (Children Asthma Control Test); CQ (Clinical Questionnaire)

The study provided a recruitment visit at time “0” in November 2019 (V0), a second visit three months later in February 2020 (V1), and a final visit after Italian Covid-19 lockdown in June 2020 (V2) (Fig. 1).

At time 0 enrolled participants were simply randomized to either the control or intervention group. All parents were equipped with a written individualized rescue management plan and formally trained for its use. In addition, the intervention group was asked to download the digital application ‘’Asmapp’’ and was instructed on its use. At V1 and V2 all children underwent medical visits, clinical history was collected, the childhood Asthma Control Test (cACT) was completed by children ≥ 4 years of age and a written clinical questionnaire (CQ) was answered by parents/caregivers. Answers to questions on acceptance and usability of the digital support were also collected from the intervention group. Finally, app monitoring data was downloaded, including the registered number of days of app use itself.

### Intervention

A dedicated smartphone application, the so-called ‘’Asmapp’’, was developed with the contribution of Chiesi Foundation. It was designed by a teamwork composed of physicians, nurses, psicologists and parents representative to monitor children’s respiratory symptoms and treatment options adopted. In addition, it provided a section on managing an acute asthma attack, accompanied by video tutorials on how to correctly administer inhaled treatments. Through a dedicated back office the study doctors could prescribe the personalized continuous therapy, but also the correct drugs to manage an asthma attack management, for each specific patient.

At V0, Asmapp was downloaded for free by participants assigned to the intervention group, and they were trained to its use. During the monitoring period, parents/caregivers filled out a daily e-Diary in the App, entering data related to their child’s symptoms, assumption of medication or additional therapies, need for extra visits or hospital admissions, and other parameters. These data were shared also with the physicians and nurses, through the back office allowing an ongoing patients’ monitoring.

A research nurse weekly checked the backup of patients’ e-diaries, going from few minutes to an hour depending on patients’ answers. The caring team got notified if a patient was experiencing frequent exacerbations, needing an earlier review. To support compliance to the e-diary those patients with a low compilation rate were phoned by the study nurse to assist them if they were experiencing difficulties with the app. Assistance was mainly operated by phone and not in person.

**Fig. 2:**
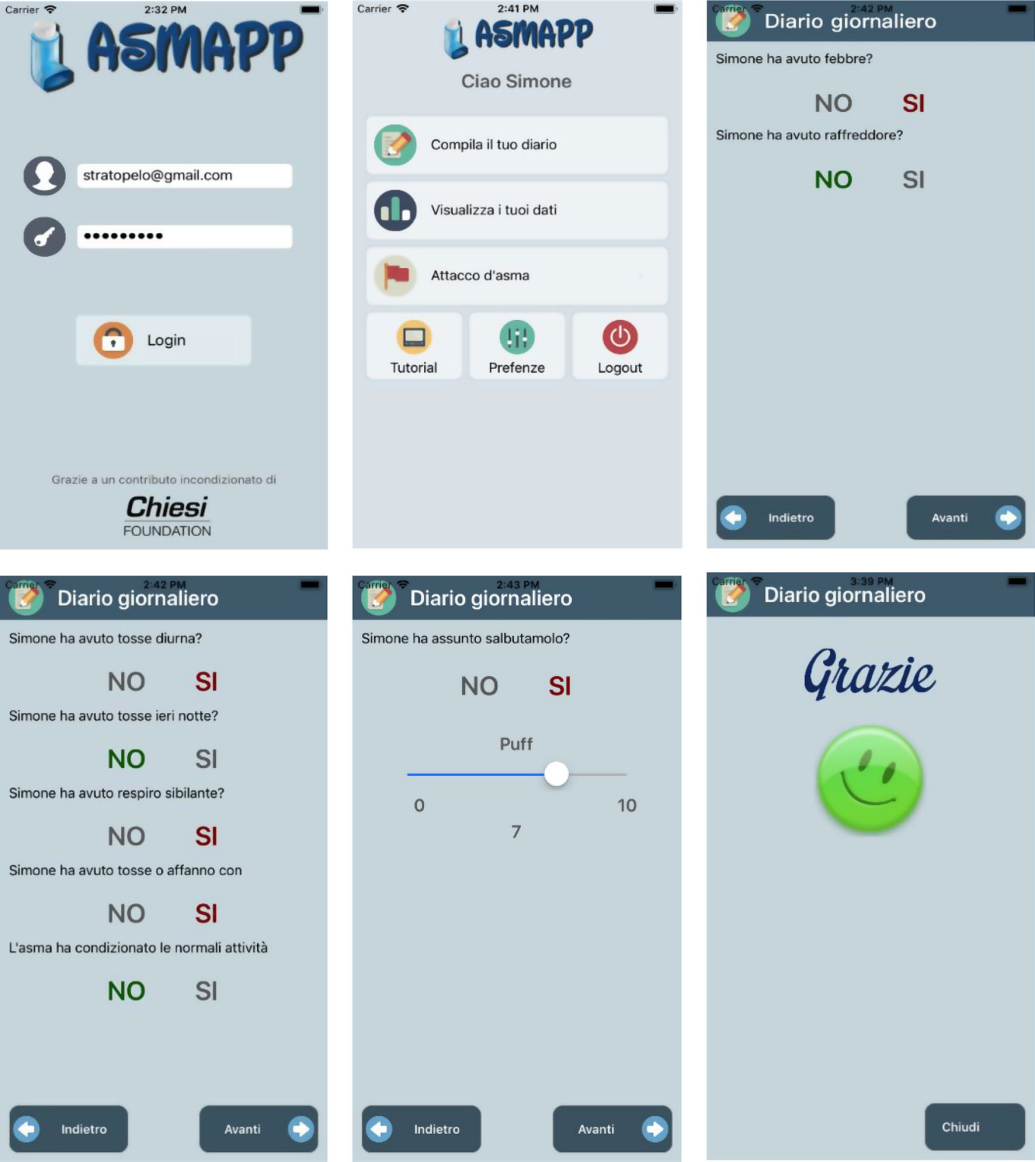
Screenshots of the App (Asmapp). Top left square: login steps; center top square: indicates different options for users (complete your diary, see your scores, asthma exacerbation, tutorial, preferences, logout); top right square: daily diary (did you have fever or cold?); bottom left square: other questions of the daily diary (did you have daily cough, night cough, wheeze, difficult breathing, symptoms that affected daily activities?); bottom middle square (did you need salbutamol?), bottom right square: thanks for completing your diary

Screenshots of the Asmapp user interface and its features can be seen in Fig. 2.

This app is not actually available from commercial stores.

### Questionnaires

The cACT is a widely validated tool designed to assess asthma control in children aged 4–11 years. It is composed of 7 questions (4 child-reported and 3 caregiver-reported) that integrate the child and caregiver’s perspectives on asthma control over the previous 4 weeks. The overall score ranges from 0 (poor control of asthma) to 27 (complete control of asthma) [16].

The CQ was specifically developed by the study authors and it was structured to collect retrospective data over the previous three months. It explored the following 14 items: episodes of wheezing, presence of cough, nighttime symptoms, asthma affecting daily activities, episodes of shortness of breath, need of salbutamol, total days of oral steroids, extra medical examinations, emergency room visits, hospital admissions, lost school days, asthma family perception, need of changing asthma therapy, use of reminders for asthma management.

Finally the intervention group received a short additional questionnaire investigating the satisfaction level of using the app.

### Study outcomes

Outcomes of the study were to explore the usability, acceptability, feasibility and efficacy of Asmapp. Usability and acceptability of the intervention was determined at follow-up from participants’ opinion about whether the app was easy to use or not and if they would recommend it (Table 1). In addition data on adherence were used to determine the feasibility of an everyday app use. Efficacy of intervention was assessed comparing the results of cACT score and CQ in both groups, searching for respiratory symptoms (Table 2).

**Table 1:** Questionnaires’ results about acceptance and usability of the app

	V1 (February 2020)	V2 (June 2020)
Participants’ opinion about App (N, %)	Problematic (2, 4.7) Clear enough (11, 25.6) Simple and intuitive (30, 69.7)	Problematic (2, 4.8) Clear enough (1, 2.4) Simple and intuitive (39, 92.8)
Participants recommending App (N, %)	Yes (41, 95.3) No (2, 4.7)	Yes (41, 97.6) No (1, 2.4)

**Table 2:** Questionnaires’ results at V1 and V2: control group versus intervention group

	V1 (November - February)			V2 (March – June)		
	Control group *N* = 42	Intervention group *N* = 43	*P* value	Control group *N* = 41	Intervention group *N*= 42	*P* value
cACT score, median (IQR) *(from 62 patients ≥4 yrs)*	23.5 (21-25)	23 (21-24)	0.47	25 (24-25)	24 (24-25)	0.13
Episodes of wheezing, N (%)						
Yes	26 (62)	25 (58)	0.72	0 (0)	0 (0)	ns
No	16 (38)	18 (42)		41 (100)	42 (100)	
Cough attacks, N (%)						
≥ Three times	8 (19)	7 (16)	0.87	0 (0)	0 (0)	0.67
Once or twice	23 (55)	26 (61)		4 (10)	3 (7)	
Never	11 (26)	10 (23)		37 (90)	39 (93)	
Nighttime symptoms, N (%)						
≥ Once a week	11 (26)	12 (28)	0.95	0 (0)	1 (2)	0.36
Once or twice	17 (41)	18 (42)		3 (7)	1 (2)	
Never	14 (33)	13 (30)		38 (93)	40 (96)	
Wheeze affecting daily activities, N (%)						
Yes	20 (48)	19 (44)	0.75	0 (0)	2 (5)	0.16
No	22 (52)	24 (56)		41 (100)	40 (95)	
Episodes of shortness of breath, N (%)						
> Twice a week	9 (21)	5 (12)	0.22	0 (0)	0 (0)	0.98
Once or twice a week	10 (24)	17 (39)		1 (2)	1 (2)	
Never	23 (55)	21 (49)		40 (98)	41 (98)	
Use of salbutamol, N (%)						
≥ Three times a day	11 (26)	10 (23)	0.60	0 (0)	0 (0)	0.15
Once or twice a day	11 (26)	14 (33)		0 (0)	0 (0)	
Twice a week	2 (5)	4 (9)		4 (10)	1 (2)	
Once a week	7 (17)	3 (7)		0 (0)	2 (5)	
Never	11 (26)	12 (28)		37 (90)	39 (93)	
Total days of oral steroids, N (%)						
≥ 5 days	14 (33)	15 (35)	0.99	1 (2)	0 (0)	0.51
< 5 days	11 (26)	11 (25)		1 (2)	2 (5)	
Never	17 (41)	17 (40)		39 (96)	40 (95)	
Extra medical visits, N (%)						
≥ Twice	18 (43)	23 (54)	0.47	0 (0)	0 (0)	0.98
Once	13 (31)	13 (30)		2 (5)	2 (5)	
Never	11 (26)	7 (16)		39 (95)	40 (95)	
Emergency Room visits, N (%)						
≥ Once a week	6 (14)	6 (14)	0.96	0 (0)	1 (2)	0.32
Never	36 (86)	37 (86)		41 (100)	41 (98)	
Hospital Admission, N (%)						
Yes	2 (5)	2 (5)	0.98	0 (0)	0 (0)	ns
No	40 (95)	41 (95)		41 (100)	42 (100)	
Lost school days, N (%)						
> 10 days	17 (40)	16 (37)	0.92	0 (0)	0 (0)	ns
6-10 days	8 (19)	8 (19)		0 (0)	0 (0)	
1-5 days	7 (17)	6 (14)		0 (0)	0 (0)	
None	10 (24)	13 (30)		41 (100)	42 (100)	
Asthma family perception, N (%)						
Not well-controlled	8 (19)	6 (14)	0.53	0 (0)	0 (0)	ns
Well-controlled	34 (81)	37 (86)		41 (100)	42 (100)	
Need of changing asthma therapy, N (%)						
Yes	5 (12)	7 (16)	0.56	5 (12)	2 (5)	0.22
No	37 (88)	36 (84)		36 (88)	40 (95)	
Use of reminders for asthma management, N (%)						
No	11 (26)	6 (14)	0.11	2 (5)	0 (0)	0.22
Not much	13 (31)	9 (21)		0 (0)	1 (2)	
Yes	18 (43)	28 (65)		39 (95)	41 (98)	

Finally, another important outcome was to assess the reliability of the clinical history information collected retrospectively at medical visits, by comparing data recorded daily with the app and the CQ in the intervention group (Table 3).

**Table 3:** Comparison among written questionnaire and app’s results from intervention group at V1 and V2

	V1 (November - February)			V2 (March – June)		
	Written questionnaire *N* = 43	App *N* = 43	*P* value	Written questionnaire *N* = 42	App *N* = 43	*P* value
Episodes of wheezing, N (%)						
Yes	25 (58)	32 (74)	= 0.1103	0 (0)	6 (14)	< 0.0120
No	18 (42)	11 (26)		42 (100)	37 (86)	
Cough attacks, N (%)						
≥ Three times	7 (17)	17 (39)	< 0.0083	0 (0)	2 (5)	< 0.0018
Once or twice	26 (60)	24 (56)		3 (7)	15 (35)	
Never	10 (23)	2 (5)		39 (93)	26 (60)	
Nighttime symptoms, N (%)						
≥ Once a week	4 (9)	2 (5)	< 0.0053	0 (0)	0 (0)	< 0.0293
Twice or three times	8 (19)	7 (16)		1 (2)	1 (2)	
Once or twice	18 (42)	32 (74)		1 (2)	9 (21)	
Never	13 (30)	2 (5)		40 (96)	33 (77)	
Wheeze affecting daily activities, N (%)						
Yes	19 (44)	27 (63)	< 0.0062	2 (5)	10 (23)	< 0.0400
No	24 (56)	16 (37)		40 (95)	33 (77)	
Episodes of shortness of breath, N (%)						
> Twice a week	5 (12)	21 (49)	< 0.0002	0 (0)	6 (14)	< 0.0219
Once or twice a week	17 (39)	9 (21)		1 (2)	3 (7)	
Never	21 (49)	13 (30)		41 (98)	34 (79)	
Use of salbutamol, N (%)						
≥ Three times a day	10 (23)	0 (0)	< 0.0001	0 (0)	0 (0)	< 0.0393
Once or twice a day	14 (33)	0 (0)		0 (0)	0 (0)	
Twice a week	4 (9)	32 (74)		1 (2)	6 (14)	
Once a week	3 (7)	8 (19)		2 (5)	6 (14)	
Never	12 (28)	3 (7)		39 (93)	31 (72)	
Total days of oral steroids, N (%)						
≥ 5 days	15 (35)	8 (18)	= 0.4029	0 (0)	1 (2)	= 0.4260
< 5 days	11 (26)	18 (42)		2 (5)	4 (9)	
Never	17 (39)	17 (40)		40 (95)	38 (89)	
Adjunctive therapy, N (%)						
Yes	7 (16)	36 (84)	< 0.0001	2 (5)	14 (33)	< 0.0010
No	36 (84)	7 (16)		40 (95)	29 (67)	
Extramedical visits, N (%)						
≥ Twice	18 (42)	17 (39)	= 0.9535	0 (0)	0 (0)	< 0.0477
Once	18 (42)	18 (42)		2 (5)	8 (18)	
Never	7 (16)	8 (19)		40 (95)	35 (82)	
Emergency room visits, N (%)						
≥ Once a week	6 (14)	26 (60)	< 0.0001	1 (2)	8 (18)	= 0.0507
Never	37 (86)	17 (40)		41 (98)	35 (82)	
Hospital admission, N (%)						
Yes	2 (5)	3 (7)	= 0.6449	0 (0)	2 (5)	= 0.1572
No	41 (95)	40 (93)		42 (100)	41 (95)	
Lost school days, N (%)						
> 10 days	16 (37)	14 (32)	= 0.1756	0 (0)	16 (37)	< 0.0001
6–10 days	8 (19)	14 (32)		0 (0)	0 (0)	
1–5 days	6 (14)	9 (22)		0 (0)	7 (16)	
None	13 (30)	6 (14)		42 (100)	20 (47)	

### Data analysis

The sample size was opportunistic as no background data were available to a power calculation. Descriptive statistics were used to analyze baseline data and primary study outcomes. Categorical data were reported as numbers (N) and frequencies (%), while for quantitative data mean/median and standard deviation (SD)/interquartile range (IQR) were calculated. Chi-squared test or Fisher test were used to evaluate the association of categorical data between groups. Taking the paired-nature of the data paired T-test for groups, Wilcoxon and McNemar Chi square were used to compare quantitative variables. A p-value < 0.05 was considered statistically significant. Statistical analyses were performed with SAS 9.4 software.

## Results

### Characteristics of the study population

Eighty-five children (43 in the intervention group and 42 in the control group) affected by recurrent wheezing attacks and asthma symptoms in between were enrolled. Of them, 48 (56%) were males, with a median age (IQ) of 5.13 (4.14–5.65) and 4.72 (3.69–5.82) years for the study and control group respectively. At visit 2 final data was collected from 83 patients, only two patients (one from each group) dropped-out the study for family problems.

The cACT score at the beginning of the study was calculated only for sixty-two (73%) patients ≥ 4 years old. The cACT score was 23 (20-24.5) and 23.5 (21–25) for the control and the intervention group, respectively, not showing any statistical difference between them.

Moreover, Table 4 with clinical data (number of wheezing exacerbations) for the previous year (2018–2019), showed no significant differences between the interventional and control group.

**Table 4:** Previous year 2018–2019 clinical data: control group versus intervention group

	(November– February)			(March – June)		
	Control group *N* = 42	Intervention group *N* = 43	*P* value	Control group *N* = 42	Intervention group *N* = 43	*P* value
> 3 wheezing attacks, N (%)	14 (33)	15 (35)	ns	9 (21)	12 (28)	ns
1 or 2 attacks, N (%)	11 (26)	8 (19)	ns	13 (31)	10 (23)	ns
Never, N (%)	17 (41)	20 (47)	ns	20 (48)	21 (49)	ns

### Adherence to digital monitoring

Parents were asked to record all child’s respiratory symptoms, as well as the daily medication. The mean (SD) number of days of follow-up was 204 (23). During this period, the average (SD) number of days with a completed symptoms diary was 123 (36) with an average (SD) adherence to e-Diary compilation of 60(15)% of the total days of follow-up (data not shown).

27 families (64%) filled in the asthma questionnaire ≥ 50% of time during the study period.

### Acceptance and usability

The acceptance and usability of the intervention have been favorable. Most patients in the intervention arm reported that the app was ‘’simple and straightforward: 70% and 92% at the first and second follow-up visits respectively. 26% (24% at V2) valuated it ‘’clear enough,‘’ while only 4.7% and 4.8% at V2 defined it ‘’problematic’’. In line with the great acceptance, the use of the app was recommended from almost all parents or caregivers (95.3% and 97.6% of cases). These results are reported in Table 1.

### Efficacy

Aim of the study was also to determine the potential role of the App for changes in clinical outcomes in comparison with the standard approach. Clinical outcomes were evaluated through measurements of the cACT test (available only for 62 patients ≥ 4 years old) and the CQ (Table 2).

All patients from both groups showed a significant clinical improvement. At V2 c-ACT score was significantly better than at V1 and CQ showed a significant drop in respiratory symptoms and need for acute treatment, similar in both groups. These results were interpreted as a significant beneficial effect from the national Covid-19 lockdown (22).

Collected data showed no statistically significant differences in each clinical outcome between the control and the intervention group, at both outpatients evaluations (V1 and V2). At V1, the cACT median score (IQR) was 23,5 (21–25) for the control group and 23 (21–24) for the intervention group (*p* = 0.47). At V2 it was 25 (24–25) and 24 (24–25), respectively (*p* = 0.13). Similarly, no significant differences were found between the two groups at follow-up visits for the following items: number of episodes of wheezing, cough attacks, nighttime symptoms, episodes of shortness of breath, need of salbutamol or oral steroids, extra medical examinations, emergency room visits, hospital admissions, lost school days, as well as in parents’ opinion about asthma affecting daily activities, asthma perception, need of changing therapy and use of reminders for asthma management. As mentioned in the limits of the study, unfortunately, our project was partially run during the unpredictable Covid pandemia which might have significantly change our efficacy results.

### Reliability of parents reports at periodical medical visits

A secondary analysis of data collected from the intervention group compared daily recorded clinical data with the results from the CQ collected at follow-up visits, as reported in Table 3.

This analysis revealed significant differences between the two monitoring methods. Specifically, at V1 and V2 the app recorded a greater number of symptoms such as cough attacks (*p* < 0.01; *p* < 0.01), nighttime symptoms (*p* < 0.01; *p* < 0.05) and episodes of shortness of breath (*p* < 0.001; *p* < 0.02), higher impact of wheeze on daily activities (*p* < 0.01; *p* < 0.04) and more use of adjunctive therapy (*p* < 0.001; *p* < 0.001) in comparison to the results from CQ. On the contrary parents reported greater use of salbutamol in the written questionnaire referring to the previous 3 months compared with what they daily completed into the app (*p* < 0.001; *p* < 0.05). Finally, no significant differences were found in the number of total days of oral steroids (*p* = 0.41; *p* = 0.43), hospital admissions (*p* = 0.64; *p* = 0.16) or lost school days (*p* = 0.17).

## Discussion

### Mobile app for preschool wheezing

The results from this study indicate that ‘’Asmapp’’ is usable, acceptable and feasible for monitoring preschool children affected by recurrent wheezing.

The adherence to the use of the app was good, reaching an average of 123/204 (60%) of all days which is acceptable for a daily questionnaire app during a long period (6 months) of follow up and 27 families (64%) filled in the asthma questionnaire ≥ 50% of time during the study period. During the study period families were worried for the Covid emergency which might have affected our results. The wide variability in adherence (SD 15%) suggests a variable family motivation which it would be important to be considered if we would consider the use of a digital monitoring system in the management of our patients. Finally, it has to be admitted that our patients accepted to take part to a clinical trial therefore, adherence to the app could be different in a context of a routine clinical practice. This hypothesis should be tested in a real-life contest.

Concerning the usability and acceptance of the device, most of the families rated its use as ‘’simple and straightforward” with a percentage increasing from the 70% to the 93% at the first and the second visit of follow-up respectively. This was reasonably due to the increased confidence gained in its use by parents, also thanks to the available assistance of researchers. Importantly, the majority of families recommended its use (95% and 98% of cases) considering it useful and reassuring to better manage their young children with recurrent problems. As previously specified in our study a research nurse weekly checked the backup of patients’ e-diaries, going from few minutes to an hour depending on patients’ answers. This is an extra amount of work to be considered for the caring team and it is probably one of the main limits to the implementation of this app in clinical practice.

### Efficacy

Similarly to previous studies in school age asthmatic children and adolescents where keeping a symptom diary was shown not to have beneficial effect on asthma control [17], none of the clinical outcomes showed a difference in the efficacy of the intervention group when compared to the controls. Voorend-van Bergen et al. few years ago showed only a greater reduction of ICS performing web-based ACTs every month rather than every 4 months, with maintained clinical control in older children [18]. In our young population, cACT score and each of the respiratory symptoms investigated as well as the medication assumed or the use of health resources were similar between the two groups, independently from the type of monitoring they used. We could have expected that the fact of having to answer every day to the app could have improved compliance to treatment with direct clinical benefits. However, this was not the case possibly due to the fact that parents of young preschoolers are themselves very careful to take care of their children health. It is also easy to suppose that participants in the trial from both groups had higher interest in following close monitoring and they have better treatment compliance than usual. However, the demonstration of similar efficacy in comparison with the standard follow-up and the great parents’ acceptability support the fact that digital monitoring could be considered as a method of remote follow-up for a few selected patients in this age group. In fact, given the costs related to disseminate as similar app and to analyze the massive amount of data produced through e-diary, we believe that it is unlikely the use of such monitoring in most of our patients. However, it could be considered for a small subgroup of children with severe and frequent wheezing attacks and poor symptoms control. Moreover, under conditions of inability or limited access to health care facilities, as was the case during the recent Covid-19 pandemic, such tools may be useful in helping parents and patients themselves in managing the condition or to be considered in a telemedicine program.

Finally, we believe that our results are strongly influenced by the national lockdown with both groups showing a significant clinical improvement and a significant drop of the use of salbutamol as needed and of oral corticosteroids, as well as the use of healthcare resources for respiratory symptoms.

### Reliability of parents’ reports

It is not self-evident that the medical history taken retrospectively during medical visits is completely representative of reality. It is important to understand if collecting patients’ medical history with a digital tool it is more reliable than with periodical face-to-face interviews [19].

In fact, our secondary outcome of this study was to assess if medical history data obtained in medical interviews every 3 months correlated with the information collected by a digital app with a daily questionnaire.

As previously showed by Okupa and coauthors [14], in older children with asthma, our data showed that parents of children with PSW have difficulties remembering a detailed clinical history referred to the previous three months and a daily questionnaire could be more reliable. We believe that this is an important finding to take into consideration as recurrence of outpatients visits for children with PSW is often every 6 months which might make medical history even less accurate.

From our data it emerged that the app reported a higher number of respiratory symptoms such as cough attacks, nighttime symptoms and episodes of shortness of breath, higher impact of wheeze on daily activities and more use of adjunctive therapy in comparison with the paper questionnaire. On the contrary parents reported greater use of salbutamol in the written questionnaire compared with what they reported in the app. Our study clearly reveals that unless parents keep a self-written diary, clinical symptoms and first line bronchodilator treatment are easily misreported. This finding it is crucial to know since continuous therapy (such as inhaled corticosteroids, montelukast etc…) is modulated mainly in relation to those anamnestic information. We believe that the use of health technology could definitively help physicians to monitor especially those children affected by difficult to control recurrent wheezing. On the contrary, total days of oral steroids, hospital admission or lost school days did not show statistically significant differences, possibly because they are given more attention by parents and difficult to be mistaken or forgotten. Given these observations, we suggest that digital monitoring with a mobile app may provide physicians a more comprehensive summary of the patients’ medical history, offering the opportunity to improve quality care by avoiding to take treatment decisions on the basis of unprecise clinical information.

This study has some limitations. First, it was partly conducted during the COVID-19 related pandemia, when preschool wheezers underwent a significant clinical improvement due to measures of social distancing and less wheezing episodes were observed. Results of efficacy and adhrence might have been different. However, this factor was not predictable and our important data on reliability of parents’ reports were probably not being affected. Second, our study was monocentric and involved only patients afferent to our Children’s Hospital in Rome, so our results might not be translated to different social and cultural contexts. Finally, another limitation is related to the use of cACT and CQ. cACT is usable for children ≥ 4 years and older, thus excluding the youngest ones, and our clinical questionnaire has not been validated but it has already been successfully adopted in previous studies [16].

## Conclusions

Our study shows that a smartphone app is usable and well accepted by parents for monitoring asthma symptoms in preschool wheeze, and it reflects daily symptoms better than retrospective investigation done in clinic. However, the asthma app did not improve clinical outcomes. This is maybe due to the fact that patients from both groups benefited from continuous inhaled corticosteroids treatment, all patients improved during COVID lockdown, and the study was not specifically powered to look at impact on clinical outcomes, given the absence of previous similar studies. Therefore, based on our results, we cannot recommend to use the app in clinical practice in children with severe PWS.

The effectiveness of this app should be explored in future studies in children with difficult-to-control PWS to assess if it has any clinical utility in daily practice especially in this selected population.

## Data Availability

The datasets used and/or analysed during the current study are available from the corresponding author on reasonable request.

## Abbreviations

App: Smartphone Application
CQ: Clinical Questionnaire
cACT: Children Asthma Control Test
ICSs: Inhaled Corticosteroids
PSW: Preschool recurrent wheeze
